# Effects of the Mediterranean Lifestyle During the COVID-19 Lockdown in Spain: Preliminary Study

**DOI:** 10.3389/fnut.2021.683261

**Published:** 2021-06-18

**Authors:** Ana Zaragoza-Martí, Miriam Sánchez-SanSegundo, Rosario Ferrer-Cascales, Eva Maria Gabaldón-Bravo, Ana Laguna-Pérez, Lorena Rumbo-Rodríguez

**Affiliations:** ^1^Nursing Department, Health Psychology and Human Behavior Research Group, Faculty of Health Sciences, University of Alicante, Institute for Health and Biomedical Research (ISABIAL), Alicante, Spain; ^2^Department of Health Psychology, Health Psychology and Human Behavior Research Group, Faculty of Health Sciences, University of Alicante, Alicante, Spain

**Keywords:** COVID-19, mediterranean diet, confinement, nutritional status, older

## Abstract

We aim to assess the beneficial effects of the Mediterranean style-diet before and after the period of confinement due to COVID-19 in a sample of 51 older patients who were part of a clinical trial of the Instituto de Investigación sanitaria y Biomédica de Alicante (ISABIAL, CEIM). Participants were randomly assigned to two conditions: experimental vs. a control group. A pre-test survey assessment was conducted before confinement, while a post-test survey was conducted after the confinement period. Adherence to Mediterranean Diet and nutritional status were evaluated through self-reported questionnaires. Individuals who initiated the Mediterranean Diet intervention program before confinement increased 3.5% their level of adherence to the Mediterranean Diet and maintained their nutritional status after the confinement. In the case of BMI, there no were statistically significant differences between groups before and after confinement. These results suggest that adherence to the Mediterranean Diet may play an important role in the establishment of appropriate dietary guidelines in confinement situations such as the COVID-19.

## Introduction

The Coronavirus disease (SARS-COV2), initially originated in the Chinese city of Wuhan in late 2019, has become a global health alarm due to its high infection potential and rapid spread. This situation has led governments of most nations around the world, including Spain, to take exceptional measures, such as the state of alarm, confining the entire population to their homes to control the progress of the contagionS (1).

The limitation of the social and personal life of the population has had a significant impact on people's health, especially in terms of habits of physical activity, psychological health levels, and eating patterns. Different studies have shown that the lockdown produced by the SARS-COV2 disease has triggered levels of anxiety, depression, perceived stress, and fear in the population, and has led to greater alterations in food and nutritional status in general (2, 3). These lifestyle changes caused by the lockdown can increase the risk of developing pathologies traditionally associated with populational sedentary lifestyles such as obesity, type II diabetes, cardiovascular disease, or chronic degenerative diseases (4). These diseases are particularly prevalent in the group of older people, who are more vulnerable, due to physiological changes caused by age in different systems such as basal metabolism, digestive system, cardiovascular system, nervous system, and motor system (5, 6).

Hence, the importance of maintaining an active lifestyle and healthy eating habits has been highlighted, especially in situations of confinement (7). In this sense, one of the best dietary patterns with the greatest health benefits is the Mediterranean Diet (MD) model. The evidence accumulated to date suggests that the MD plays a protective role in the prevention of numerous pathologies. The MD is characterized by being rich in fruits and vegetables, legumes, whole grains, nuts, and olive oil; because it is moderate in dairy, fish and lean meats and because it includes occasional consumption of red meats and derivatives and processed foods. Besides, the MD pattern is also characterized by having a social nature, that is, eating at the table with family, sharing experiences, respecting local gastronomy and seasonal products, as well as promoting an active lifestyle (8).

The benefits of the MD have been highlighted in numerous individual studies and meta-analyses showing that it reduces mortality rates by 8%, the risk of developing cardiovascular disease by 10%, and other diseases by 4% (9). The MD has also been shown to have a protective effect against the development of some cancers and the delay in the onset of neurodegenerative diseases associated with aging (10). The MD has been considered to be the most beneficial diet due to anti-inflammatory and antioxidative properties, particularly during aging. For example, the effect of the Mediterranean Diet on Metabolic Health has been found in a recent meta-analysis of controlled trials of 36,983 participants, which showed the beneficial changes in 18 of 28 metabolic syndrome components (11). Also, a recent meta-analysis of cross-section studies comparing 13,733 participants from 5 countries has shown a positive association between adherence to the MD and longer telomere length. It has been suggested that telomere shortening is the primary cause of aging. Thus, MD may play an important role in the preservation of telomere length and longevity (12).

Thus, following a healthy diet pattern such as the Mediterranean diet, is important, given that the levels of gene expression of cytokinins can be modified according to the foods that are ingested and they may be able to modulate the inflammatory processes, oxidative stress, and telomere length related which have been associated with aging and multiples diseases including COVID-19. There is evidence showing that the key to the Mediterranean diet in modulating the inflammatory response is due to the MD is characterized by low cholesterol levels, high levels of antioxidants from fruits and vegetables and high content of monounsaturated fatty acids and polyunsaturated precedents from olive oil, nuts, and oily fish (13).

From this perspective of promoting Mediterranean health and lifestyle, the objective of this work was to determine the degree of adherence to the MD and the changes in nutritional status before and after the lockdown in a sample of older citizens residing in the Spanish Mediterranean. To date, the PREDIMED multicenter, randomized prevention trial is the most important study conducted in Spain that demonstrates the benefits of the MD diet on health and in particular in cardiovascular disease (14). A previous study conducted in Spain has demonstrated that the COVID-19 confinement in Spain has led to the adoption of better healthy dietary habits, including a higher adherence to the MedDiet (15). However, a more recent work has also found that the consumption of unhealthy food has also increased during the pandemic situation caused by the COVID-19 infection in Spain (4).

Given the previous positive findings of the MD we hypothesize that individuals who initiated an intervention program based on the benefits of the MD before the lockdown will show adequate adherence to the MD and maintenance of their nutritional status. To confirm this hypothesis, we evaluated a sample of 51 patients who were part of the clinical trial of the Alicante Institute for Health and Biomedical Research (ISABIAL, CEIM).

## Materials and Methods

### Sample and Procedure

The sample included 51 older people from the Mediterranean city of Alicante (Spain). Participants ranged in age from 60 to 74 years (*M* = 65, *SD* = 4, 67). Inclusion criteria were: (1) being over 60 years of age and having attended the scheduled consultation of the health care professional of the health center, (2) having a Body Mass Index (BMI) >25 Kg/m^2^. Exclusion criteria were: patients with a score of 3 or more errors (if they had studies) in the Pfeiffer's Short Portable Mental Status Questionnaire which evaluates the cognitive deterioration by means of 10 questions (i.e., who is the current president?) (16) and four or more errors (no studies), having reported reading and writing difficulties, having followed dietary-nutritional treatment supervised by a nutritionist over the past year, and being considered too fragile according to medical or nursing criteria. Participants were randomly assigned to two conditions: experimental vs. control wait-list group. Ten participants were excluded from the initial sample. Five participants (2.5%) were excluded due to having followed a dietary treatment over the past year, 2 (1%) participants scored above three errors in the Pleiffer‘s questionnaire and 3 (1.5%) declined participation. The remaining 51 participants were assigned to the experimental group (*n* = 25; 96% females; 4% males) or the control group (*n* = 26; 84.6% females; 15.4% males). During the follow-up (Time 2 and Time 3), two participants from the experimental group dropped out of the intervention program and were classified as non-completers, mostly because they refused to be part of the follow-up data collection.

The study was approved by the Ethics Committee of the Instituto de Investigación Sanitaria y Biomédica de Alicante [ISABIAL (Health and Biomedical Research Institute of Alicante)] (CEIm: PI2019/057). After being informed about the study, their voluntary participation, and that they could withdraw from the study with no consequences, participants were requested to participate. Consents were obtained from the entire sample. Several additional attempts were made by the project staff to engage participants who did not return a signed form.

### Experimental Design

Participants were recruited from the Department of Health Alicante—General Hospital (Spain). Demographic data, including age, sex, nationality, employment status, financial income, weight, size, BMI, and hip-waist perimeter were gathered from the participants. All participants completed a pre-/post-test battery of questionnaires which included adherence levels to the MD, anthropometric data, lifestyle, and physical activity. For the experimental group, the pre-test battery was followed by the implementation of the intervention program over the subsequent 6 months, between September 2019 and June 2020 and 3 months of follow-up. The program consisted of nine 1-h individual sessions and six 2-h group sessions distributed across 6 months. A cut-off ≥ 7 sessions of the total number of sessions was used to classify participants as completers (representing at least 80% attendance of the program).

### Intervention Program

A multi-component intervention was performed for 6 months and 3 of follow-up. Participants in the experimental group received personalized training for weight management, food education, psychological support, and self-care recommendations. During the individual sessions, each participant received a menu adapted to their needs based on the MD. Besides, three group sessions were conducted. During the group sessions, general training in MD, nutritional labeling, and healthy recipes were provided Psychological support sessions throughout the intervention included issues related to self-control, anxiety management, emotional feeding, achievements, and difficulties encountered during treatment, review of weekly action plans, relaxation techniques, consolidation of the new image, cognitive distortions, change management, coping with uncertainty, and motivation to change. These sessions were conducted by highly qualified psychology professionals. For physical activity, at least 150 min per week was recommended, following the recommendations of the World Health Organization (WHO). In these sessions, in addition to the practice of physical exercise, training was included in sedentarism (epidemiology and consequences), health benefits of physical exercise, types of physical exercise, and community benefits and resources for the practice of physical exercise. The intervention program was conducted from September 2019 to March 2020. The follow-up was conducted from March to June 2020 through several methods: telephone, whatsApp^TM^, mail and a short electronic questionnaire which was shared *via* Google survey platform. Also, all participants who received the intervention were invited to participate several online courses about healthy diet during confinement within the Aula de la Salud of the University of Alicante (see https://mayoresalud.ua.es/formacion/#alimentacion).

### Measures

#### Eating Habits

To determine the degree of adherence to the MD, we employed a specific short questionnaire of 14 items validated for the Spanish population and used by the Mediterranean Diet Prevention group (PREDIMED) (17). To obtain the score, each item with a positive connotation for the MD is assigned a value of +1 and, if the item has a negative connotation, it receives a value of −1. From the sum of the values obtained in the 14 items, the degree of adherence is determined, establishing two levels, such that if the total score is ≥9, the score reflects a good level of adherence and if the total sum is <9, the score shows low adherence. To determine participants' eating habits, a dietary interview was prepared, following the guidelines of the Spanish Society of Dietitians-Nutritionists.

#### Sociodemographic and Clinical Variables

An *ad hoc* questionnaire was used to collect sociodemographic, clinical, and lifestyle data. The sociodemographic data contemplated in our study were age, sex, marital status, years of schooling, and place of residence. The clinical variables studied were systolic and diastolic blood pressure, weight, and height. Finally, the lifestyle variables studied were actual alcohol consumption (never or yes on a regular basis) tobacco use (never or yes on a regular basis), and physical activity (hours/weeks).

#### Anthropometric Variables

Standardized methods were used to measure anthropometric data. Bodyweight was measured using a vertical mechanical scale with SECA700® sliding weights, with an accuracy of 100 g. Height was measured with an accuracy of 0.2 cm, using the vertical meter. With the data of weight in kg and height in cm, the BMI was calculated (BMI = weight/height^2^; that is, Kg/m^2^). BMI was interpreted using the WHO classification (BMI <18.8 = low weight; BMI between 18.5 and 24.99 = normal weight; BMI between 25 and 29.9 = overweight; and BMI > 30 = obesity). Body perimeters were measured in triplicate (later obtaining the mean) with an extendable tape measure. The waist perimeter measurement was performed below the rib cage and above the navel (the narrowest waist circumference). The hip perimeter was taken horizontally at the maximum extension area of the buttocks (largest posterior protrusion). With the result of the two measurements, the waist-hip index (ICC = waist/hip) was calculated. To assess the presence of cardiovascular risk, participants were classified according to the result of the waist-hip index. In the case of women, cardiovascular risk was determined when the index was 0.85 or more and, for men, when it was 0.94 or more.

### Statistical Analysis

Descriptive statistics were used to define the proportion of responses for each question and the total distribution of the questionnaire. The normality of the data was confirmed using the Shapiro-Wilks *W*-test. Values were reported as means and percentage. To assess the intervention effect before and after the confinement period, two independent Generalized Estimating Equations (GEE) for the dichotomous variables “adherence to MD” and “obesity” were applied using the binomial link function with an exchangeable correlation structure and adjusted by age. These analyses were performed using the geepack R program v.4.0.2. Furthermore, the percentage change between scores from T1 to T2 was calculated following this formula: [(T2-T1/T1)]^*^100. These analyses were performed using IBM SPSS, Statistics for Windows, Version 24.0, considering *p* < 0.05 as significant.

## Results

All participants in the study had Spanish nationality and lived in the province of Alicante. The average age of the control group was 66.60 years vs. 63.44 years of the experimental group. Regarding marital status, there were no single participants, and more than 65% of the sample, both in the experimental group and the control group, was married. In the case of employment, 40% of the participants in the experimental group were active full-time compared to 7.7% of the control group. Regarding coexistence, more than 70% of the participants lived with their partner, and none of the participants lived in residences or in the home of other relatives. All the other sociodemographic and lifestyle variables are shown in Table 1.

**Table 1:** Sociodemographic characteristics of the participants.

	**Control group**	**Experimental group**	**Total**	***P***
	***N* = 26**	***N* = 25**		
**Age**	66.69 ± 5.14	63.44 ± 3.40	65.1 ± 4.67	0.01
**Sex**				0.61
Female	24 (92.3)	22 (88.0)	46 (90.2)	
Male	2 (7.7)	13 (12.0)	15 (9.8)	
**Marital status**				0.54
Married	17 (65.4)	19 (76.0)	36 (70.6)	
Others	9 (34.6)	6 (24.0)	15 (29.4)	
**Nationality**				
Spanish	26 (100.0)	25 (100.0)	51 (100.0)	
**Occupational situation**				0.12
Full-Party time/employee	5 (19.2)	11 (44.0)	16 (31.3)	
Retired	16 (61.6)	9 (36.0)	25 (49.0)	
Other	5 (2, 18)	5 (20.0)	10 (19.7)	
**Coexistence**				
At home alone	6 (23.1)	3 (12.0)	9 (17.6)	0.58
At home with my partner	19 (73.1)	21 (84.0)	40 (78.4)	
Others	1 (3.8)	1 (4.0)	2 (3.9)	
**Educational level**				0.19
No studies	1 (3.8)	1 (4.0)	2 (3.9)	
Primary studies	11 (42.3)	4 (16.0)	15 (29.4)	
Middle studies	10 (38.5)	12 (48.0)	22 (43.1)	
Higher education	4 (15.4)	8 (32,0)	12 (23.5)	
**Income**				0.25
<1000€	13 (50.0)	8 (32.0)	21 (42.0)	
>1000€	13 (50.0)	17 (68.0)	30 (58.0)	
**Tobacco use**				0.66
Yes	2 (7.7)	3 (12.0)	5 (9, 8)	
No tobacco use	24 (92.3)	22 (88.0)	46 (90,2)	
**Alcohol consumption**				0.78
Yes	12 (46.2)	13 (52.0)	25 (49.0)	
Doesn't drink alcohol	14 (53.8)	12 (48.0)	26 (51.0)	

As displayed in Table 2, there were a significant effect of intervention in the experimental group after adjusted for age (OR 9.8, 95% CI 3.61-26.78), *p* < 0.001. However, there were no significant effect of the intervention regarding the BMI, again after adjusted for age (OR 0.90, 95% CI 0.38-2.14), *p* < 0.812.

**Table 2:** GEE models for high adherence to MD and for Obesity.

		**OR**	**IC 95%**	***P*-value**
**High adherence to MD**				
GROUP	Control	1		
	Experimental	9.83	(3.61–26.78)	<0.001
Age	(years)	1.01	(0.92–1.12)	0.779
**Obesity**				
GROUP	Control	1		
	Experimental	0.90	(0.38–2.14)	0.812
Age	(years)	0.96	(0.87–1.05)	0.371

Table 3 shows adherence to MD and nutritional status before and after the lockdown, taking into account the distribution into the experimental group or the control group. Regarding adherence to MD, in the control group, no variation was observed before and after the lockdown, but in the case of the experimental group, there was a significant improvement of 3.5% of percentage of change to adherence to MD. Concerning nutritional status, the experimental there were few variations after the lockdown with no significant effect before and after lockdown.

**Table 3:** Mean of percentage change by groups in the score of Adherence to the MD and nutritional status before and after the lockdown.

	**Mean (SD) before lockdown**	**Mean (SD) after lockdown**	**Percentage change**
**Adherence to MD (PREDIMED total score)**			
Control group	7.88 ± 1.75	7.88 ± 2.75	0.00%
Experimental group	9.00 ± 1.68	9.32 ± 1.68	3.50%
**BMI**			
Control group	31.98 ± 3.88	32.05 ± 3.95	0.21%
Experimental group	31.05 ± 4.38	31.08 ± 4.44	0.09%

*MD, Mediterranean diet; BMI, body mass index*.

Table 4 shows the dietary behavior of participants during the lockdown. We observed that more than 60% of the control group participants ingested more food during the lockdown compared to 48% of the experimental group. Concerning the way of cooking, we found that the experimental group changed the way they cooked to a healthy cooking style in 84% of cases. Finally, concerning snacking throughout the day, we observed that more than 50% of the participants in both groups performed some type of snacking, in 56% of the cases due to anxiety in the experimental group vs. 34.6% in the control group.

**Table 4:** Eating behavior during the lockdown in the control group and the experimental group.

	**Control group**		**Experimental group**	
	**Yes**	**No *n* (%)**	**Yes *n* (%)**	**No *n* (%)**
	***n* (%)**	***n* (%)**	***n* (%)**	***n* (%)**
Have you eaten more food than normal during the lockdown?	16 (61.5)	10 (38.5)	12 (48.0)	13 (52.0)
If yes, was it healthy food?	15 (57.7)	11 (42.3)	16 (64.0)	9 (36.0)
Have you changed the way you cook during the lockdown?	5 (19.2)	21 (80.8)	8 (32.0)	17(68.0)
If so, has it been healthy?	22 (84.6)	4 (15.4)	21 (84.0)	4 (16.0)
Have you snacked throughout the day during the lockdown?	14 (53.8)	12 (46.2)	15 (60.0)	10 (40.0)
If yes, was it due to anxiety?	9 (34.6)	17 (65.4)	14 (56.0)	11 (44.0)

## Discussion

The COVID-19 lockdown has significantly affected the population's dietary patterns and physical activity, with most studies showing that it is strongly associated with increased weight gain and BMI. These negative effects have been associated with high levels of isolation, fear, anxiety, and depression generated by the current emergency crisis. However, while lockdown is a necessary measure to protect public health, it is also necessary to provide global interventions to mitigate the negative lifestyle patterns that are commonly manifested during confinement periods, particularly among older people, who are more vulnerable to adverse outcomes (18).

In the current study, we examined the changes in nutritional status in a sample of older residents of the Spanish Mediterranean area before and after confinement due to COVID-19. Also, we examined how the adherence to the Mediterranean diet may play a protective role in nutritional status during the lockdown. As we hypothesized, individuals who began an intervention program based on the Mediterranean diet before confinement could maintain their nutritional status and increase their level of adherence to the MD after confinement. Similar to our findings, the beneficial effects of the MD has been recently found in a Spanish study examining dietary changes of Spanish adult population during the COVID. Authors found that the adherence to the MD increased by 0.8 points during the three first weeks of confinement period as compared with previous habits (15).

Most studies have reported that diets that reduce calorie intake for weight loss are frequently unbalanced, given that these diets are mainly aimed at the reduction of intake of carbohydrates and an increase of fats and proteins (19). In contrast, the MD can help in weight control and generate a balanced nutritional status, as it has been reported as one of the best models of healthy eating due to its contribution to good health (20). Mediterranean diet, which has been considered as rich in nutrients with antioxidant and anti-inflammatory effect may have health benefits for older people, including the prevention of neurodegenerative disease and the modification of the immune and inflammatory response (21). Mediterranean diet has been associated with beneficial effects on weight circumference, blood lipids, and blood pressure (13). It also plays an important role in bone mineralization, which is particularly significant in lockdown situations due to the reduction of physical activity and mobility. Unfortunately, nutritional status as a preventive measure is not well-considered in the current emergency crisis (22, 23).

We have reported how educational interventions based on the beneficial effects of the Mediterranean diet can create an environment in which people can evaluate the positive outcomes of following the recommended lifestyle patterns of (24) as well as receiving guidance to maintain healthy choices under adverse situations such as the COVID-19. This set of circumstances is particularly significant in older people, given that body composition tends to change with aging, and the lockdown can lead to a loss of muscle mass and functional mobility due to lack of physical activity and weight gain. Additional studies are needed to support the current findings in Spain and worldwide, given the current emergency crisis due to COVID-19.

There are several limitations in the current study that suggest areas for future research. Firstly, we used a small sample size conducted in a single city in Spain, and therefore, researchers and interventionists must use caution when generalizing the findings to other areas in Spain or Europe. Second, measures were estimated based on self-reported data. Thus, some patients may underestimate their responses to the questionnaire. Also, many of the variables assessed in this study were derived from single-item measures, a common technique in epidemiological research. Therefore, future studies could be addressed using validated multi-item measures of the constructs and including possible determinants of the MD and nutritional status. Finally, future research could evaluate the effectiveness and feasibility of culturally tailored interventions for older Spanish citizens and families, which target some of the variables associated with nutritional status found in this study. Despite these limitations, this study provides solid evidence of the effectiveness of the MD on the maintenance of weight and nutritional status of the older population. Future research should seek to replicate the study in Spain and also explore the impact of the MD in clinical and mental health outcomes.

## Conclusion

In conclusion, adherence to the Mediterranean-style diet can play an important role in the establishment of appropriate dietary guidelines in lockdown situations. To achieve nutritional and health improvements global interventions are needed to mitigate the negative lifestyle patterns that are commonly shown during confinement periods, particularly among older people, who are more vulnerable to adverse outcomes. Research of the beneficial effects of MD has shown that the MD can contribute to the prevention of physical and neurodegenerative diseases and also to the modification of the immune and inflammatory response. Educational strategies of recommended intake should be specific, measurable, achievable, realistic, and timely (SMART), based on the effects of the MD on health, and they should be accompanied by physical activity to create an environment in which people can evaluate the positive outcomes.

## Data Availability Statement

The original contributions presented in the study are included in the article/supplementary material, further inquiries can be directed to the corresponding author/s.

## Ethics Statement

The studies involving human participants were reviewed and approved by Instituto de Investigación Sanitaria y Biomédica de Alicante (ISABIAL) (CEIm: PI2019/057). The patients/participants provided their written informed consent to participate in this study.

## Author Contributions

AZ-M and MS-S: conceptualization, writing—original draft preparation, and writing—review and editing. AZ-M: methodology, software, formal analysis, resources, data curation, project administration, and funding acquisition. AZ-M, MS-S, and LR-R: validation. AZ-M, LR-R, RF-C, AL-P, and EG-B: research, visualization, and supervision. All authors have read and agreed to the published version of the manuscript.

## Conflict of Interest

The authors declare that the research was conducted in the absence of any commercial or financial relationships that could be construed as a potential conflict of interest.

## References

[1] Bao Y Sun Y Meng S Shi J Liu L. 2019-Ncov epidemic: address mental health care to empower society. Lancet. (2020) 22:e37-8. 10.1016/S0140-6736(20)30309-3 32043982 PMC7133594

[2] Abbas AM Mohsen Kamel M. Dietary habits in adults during quarantine in the context of COVID-19 pandemic. Obes Med. (2020) 19:100254. 10.1016/j.obmed.2020.100254 32427142 PMC7227490

[3] Pellegrini M Ponzo V Rosato R Scumaci E Goitre I Benso A. Changes in weight and nutritional habits in adults with obesity during the “lockdown” period caused by the COVID-19 virus emergency. Nutrients. (2020) 12:2016. 10.3390/nu12072016 32645970 PMC7400808

[4] Sánchez-Sánchez E Ramírez-Vargas G Avellaneda-López Y Orellana-Pecino JI García-Marín E Díaz-Jimenez J. Eating habits and physical activity of the Spanish population during the COVID-19 pandemic period. Nutrients. (2020) 12:E2826. 10.3390/nu12092826 32942695 PMC7551353

[5] Zaragoza-MartíAFerrer-CascalesRHurtado-SánchezJALaguna-PérezACabañero-MartínezMJ. Relationship between adherence to the Mediterranean diet and health-related quality of life and life satisfaction among older adults. J Nutr Health Aging. (2018) 22:89-96. 10.1007/s12603-017-0923-229300427

[6] Ruiz-Roso MB Knott-Torcal C Matilla-Escalante DC Garcimartín A Sampedro-Nuñez MA Dávalos A. COVID-19 lockdown and changes of the dietary pattern and physical activity habits in a cohort of patients with Type 2 diabetes mellitus. Nutrients. (2020) 12:2327. 10.3390/nu12082327 32759636 PMC7468739

[7] Martinez-Ferran M Guía-Galipienso F Sanchis-Gomar F Pareja-Galeano H. Metabolic impacts of confinement during the COVID-19 pandemic due to modified diet and physical activity habits. Nutrients. (2020) 12:1549. 10.3390/nu12061549 32466598 PMC7352228

[8] Zaragoza-Martí A Cabañero-Martínez MJ Hurtado-Sánchez JA Laguna-Pérez A Ferrer-Cascales R. Evaluation of Mediterranean diet adherence scores: a systematic review. BMJ Open. (2018) 1–8. 10.1136/bmjopen-2017-019033 29478018 PMC5855302

[9] Sofi F Macchi C Abbate R Gensini GF Casini A. Mediterranean diet and health status: an updated meta-analysis and a proposal for a literature-based adherence score. Public Health Nutr. (2014) 17:2769-82. 10.1017/S1368980013003169 24476641 PMC10282340

[10] Sofi F Abbate R Franco Gensini G Casini A. Accruing evidence on benefits of adherence to the Mediterranean diet on health: an updated systematic review and meta-analysis. J Clin Nutr. (2010) 92:1189-96. 10.3945/ajcn.2010.29673 20810976

[11] Papadaki A Nolen-Doerr E Mantzoros CS. The effect of the Mediterranean diet on metabolic health: a systematic review and meta-analysis of controlled trials in adults. Nutrients. (2020) 12:3342. 10.3390/nu12113342 33143083 PMC7692768

[12] Canudas S Becerra-Tomás N Hernández-Alonso P Serena Galié Leung C Crous-Bou M. Mediterranean diet and telomere length: a systematic review and meta-analysis. Adv Nutr. (2020) 11:1544-54. 10.1093/advances/nmaa079 32730558 PMC7666892

[13] Maiorino MA Bellastella G Longo M Caruso P Esposito K. Mediterranean diet and COVID-19: hypothesizing potential benefits in people with diabetes. Front Endocrinol. (2020) 16:574315. 10.3389/fendo.2020.574315 33042027 PMC7525209

[14] Martínez-González MA Salas-Salvadó J Estruch R Corella D Fitó M. Benefits of the Mediterranean diet: insights from the PREDIMED study. Prog Cardiovasc Dis. (2015) 58:50-60. 10.1016/j.pcad.2015.04.003 25940230

[15] Rodríguez-Pérez C Molina-Montes E Verardo V Artacho R García-Villanova B Guerra-Hernández EJ. Changes in dietary behaviours during the COVID-19 outbreak confinement in the Spanish COVIDiet study. Nutrients. (2020) 12:1730. 10.3390/nu12061730 32531892 PMC7353108

[16] Martínez de la Iglesia J Dueñas Herrero R Onís Vilches MC Aguado Taberné C Albert Colomer C Luque Luque R. Cross-cultural adaptation and validation of Pfeiffer's test (Short Portable Mental Status Questionnaire [SPMSQ]) to screen cognitive impairment in general population aged 65 or older. Med Clin. (2001) 117:129–34. 10.1016/s0025-7753(01)72040-4 11472684

[17] Martínez-González MA Fernández-Jarne E Serrano-Martínez M Gomez-García E. Development of a short dietary intake questionnaire for the quantitative estimation of adherence to a cardioprotective Mediterranean diet. Eur J Clin Nutr. (2004) 58:1550–2. 10.1038/sj.ejcn.1602004 15162136

[18] Achraf Ammar A Brach M Trabelsi K Chtourou H Boukhris O Masmoudi L. Effects of COVID-19 home confinement on eating behaviour and physical activity: results of the ECLB-COVID19 international online survey. Nutrients. (2020) 12:1583. 10.3390/nu12061583 32481594 PMC7352706

[19] Ortega RM. Importance of functional foods in the Mediterranean diet. Public Health Nutr. (2006) 9:1136-40. 10.1017/S1368980007668530 17378953

[20] Serra-Majem L Roman B Estruch R. Scientific evidence of interventions using the Mediterranean diet: a systematic review. Nutr Rev. (2006) 64(suppl_1): S27-47. 10.1111/j.1753-4887.2006.tb00232.x 16532897

[21] Stark AH Madar Z. Olive oil as a functional food: epidemiology and nutritional approaches. Nutr Rev. (2002) 60:170–6. 10.1301/002966402320243250 12078915

[22] Azzolino D Saporiti E Proietti M Cesari M. Nutritional considerations in frail older patients with COVID-19. J Nutr Health Aging. (2020) 24:696-8. 10.1007/s12603-020-1400-x 32744563 PMC7256177

[23] Scarmozzino F Visioli F. Covid-19 and the subsequent lockdown modified dietary habits of almost half the population in an Italian sample. Foods. (2020) 9:675. 10.3390/foods9050675 32466106 PMC7278864

[24] Mazloomy-Mahmoodabad SS Navabi ZS Ahmadi A Askarishahi M. The effect of educational intervention on weight loss in adolescents with overweight and obesity: application of the theory of planned behavior. ARYA Atheroscler. (2017) 13:176-83. 29147128 PMC5677321

